# Most commensally bacterial strains in human milk of healthy mothers display multiple antibiotic resistance

**DOI:** 10.1002/mbo3.618

**Published:** 2018-03-25

**Authors:** Mao‐Sheng Huang, Ching‐Chang Cheng, Shu‐Ying Tseng, Yi‐Ling Lin, Hui‐min Lo, Po‐Wen Chen

**Affiliations:** ^1^ Department of Medicine Laboratory St. Mary's Hospital Luodong Yilan Taiwan; ^2^ Laboratory Animal Service Center Office of Research and Development China Medical University Taichung Taiwan; ^3^ Department of Veterinary Medicine National Chung Hsing University Taichung Taiwan; ^4^ Department of Obstetrics Central St. Mary's Hospital Luodong Yilan Taiwan; ^5^ Department of Nursing St. Mary's Junior College of Medicine Nursing and Management Yilan Taiwan

**Keywords:** antibiotic resistance, commensal bacterium, human milk, total bacterial counts

## Abstract

Recent reports have shown that food‐borne or commensal bacteria can function as reservoirs of antibiotic resistance. However, the antibiotic susceptibility of bacterial isolates of most milk samples or the total bacterial counts (TBC) in human milk from healthy donors, are not fully understood in Taiwan. Thus, five healthy mothers were randomly recruited each month, and totally 30 mothers without any symptoms of infection were recruited over 6 months. Milk samples were then harvested and analyzed immediately after collection. The antibiotic susceptibility was analyzed in bacteria isolated from milk samples using nine clinically relevant antibiotics, such as oxacillin, ampicillin, cephalothin, amoxicillin, ciprofloxacin, erythromycin, clindamycin, gentamicin, and oxytetracycline. The *Staphylococcus* strains (48 isolates) found in milk resisted to 48.6 ± 20.1% selected antibiotics. *Streptococcus*‐related isolates (8 isolates) exhibited resistance to 41.7 ± 26.4% selected antibiotics. *Acinetobacter* isolates (5 isolates) were resistant to 66.7 ± 13.6% antibiotics, and *Enterococcus* isolates (5 isolates) were resistant to 73.3 ± 6.1% tested antibiotics. *Rothia*‐related isolates (4 isolates) were resisted to 58.2 ± 31.9% of tested antibiotics. In contrast, *Corynebacterium* isolates (5 isolates) were sensitive to 66%–100% of selected antibiotics. Furthermore, the TBC ranged from 40 to 710,000 CFU/ml, implying a wide spectrum of bacteria in milk from healthy mothers. Despite this, all milk donors were healthy during sampling, and they did not show any symptoms related to mastitis or subclinical mastitis. According to the previously described TBC criteria for the use of donated human milk, only 73% of the current milk samples could be accepted for the milk bank. In conclusion, the majority of the isolated bacterial strains from current human milk samples are multiresistant strains. In milk samples for preterm infants or milk banks, higher TBC levels or potentially antibiotic‐resistant bacteria in some milk samples have supported people using approaches to disinfect human milk partially.

## 1 INTRODUCTION

Human milk is generally accepted to be the ideal food for babies, and it is a rich fluid that contains essential nutrients, such as various bioactive compounds, proteins, carbohydrates, immune cells, and immunoglobulins, which can provide sufficient nutritional and protective requirements for infants (Petherick, 2010; Walker, 2010). It can also contain various commensal or lactic acid bacteria strains (Jeurink et al., 2013; Martin et al., 2003; Heikkila & Saris, 2003). These commensal bacteria in milk can be ingested by infants, supporting that these commensal bacteria in milk may play a role in establishing the microbiota in the infant gut (Fernandez et al., 2013; Solis, de Los Reyes‐Gavilan, Fernandez, Margolles, & Gueimonde, 2010). Several recent reports have also shown that food‐borne or commensal bacteria can function as reservoirs of antibiotic resistance genes, which are similar to those found in pathogenic bacterial strains (Devirgiliis, Zinno, & Perozzi, 2013; Mathur & Singh, 2005; White, Zhao, Simjee, Wagner, & McDermott, 2002). For example, *Enterococcus* spp. collected from milk of healthy donors, such as healthy women, pigs, dogs, sheep, and cats, have been found to exhibit resistance to various clinically relevant antibiotics, including gentamicin, streptomycin, quinupristin/dalfopristin, tetracycline, and chloramphenicol (Jimenez et al., 2013). Moreover, *Staphylococcus epidermidis* isolated from human milk of healthy mothers also shows resistance to gentamicin, tetracycline, erythromycin, clindamycin, and vancomycin (Begovic et al., 2013; Delgado et al., 2009). Another study demonstrated that several *Enterococcus faecalis* and *E. faecium* isolates from human milk contained virulence genes and antibiotic resistance that could serve as a reservoir of antibiotic resistance in offspring (Jimenez et al., 2013). In our pilot study in Taiwan, most bacteria isolated from human milk of 19 healthy donors also displayed mild to strong antibiotic resistance. For example, milk‐isolated *Staphylococcus* isolates (22 strains) were resistant to 25%–100% of antibiotics, whereas *Streptococcus* isolates (three strains) were resistant to 33%–77% of antibiotics. Members of the genus *Enterococcus* (five strains) were resistant to 33%–88% of selected antibiotics (Chen, Tseng, & Huang, 2016). However, only 19 healthy donors had been recruited in this pilot study, and the antibiotic susceptibility of bacterial isolates of most human milk samples are not fully understood in Taiwan. Therefore, this study aimed to dissect the antibiotic susceptibility patterns among commensal bacteria isolated from newly 30 human milk samples of healthy mothers. At this time, about five healthy mothers without any symptoms of infection and clinical mastitis attending hospital were voluntarily and randomly recruited each month, and totally 30 milk samples were collected aseptically during a period of 6 months. Since cesarean section (CS) involves antibiotics exposure, it would be interesting to study the data in comparison between CS and vaginal delivery (often without antibiotic use during delivery). Therefore, milk samples harvested from mothers after normal spontaneous delivery (NSD) and those after CS delivery were also recorded and dissected. On the other hand, several regulations or criteria toward the total bacterial counts (TBCs) have been employed to evaluate the milk quality under the conditions of human milk sharing or milk samples collected by milk banks (Balmer & Wharton, 1992). However, this information is still vague in most human milk samples collected from healthy mothers in Taiwan. Then, we also evaluated the characteristics of milk samples by determining the TBCs levels. Human milk samples are shared between babies by the help of milk banks in Taiwan. Our findings are expected to help evaluate the TBCs and antibiotic susceptibility levels in human milk from healthy donors.

## 2 MATERIALS AND METHODS

### 2.1 Ethics, consent, and permissions

This study was approved by the Institutional Review Board (IRB) of Saint Mary's Hospital, Lundong (IRB104011), and informed consent was obtained from all participants.

### 2.2 Milk sampling

Five healthy mothers without any symptoms of infection and clinical mastitis attending Saint Mary's Hospital Lundong were voluntarily and randomly recruited each month, and totally 30 different milk samples were collected over a 6‐month period. Milk samples harvested from mothers after NSD and those after CS delivery were also recorded. These milk samples were collected by manual expression using sterile gloves after cleaning the breasts with sterile warm water or saline according to previous reports (Chen et al., 2016; Albesharat, Ehrmann, Korakli, Yazaji, & Vogel, 2011). Briefly, the first 1–2 ml milk was discarded to avoid possible contamination from the environment. After that, about 10–15 ml milk was collected in sterile tubes. The collected milk samples were transferred and analyzed at the lab immediately as indicated below. Collectively, each milk donor provided one sample, and we further divided the samples into several parts after mixing well. One part was used for aerobic TBCs test, bacterial isolation and antibiotic‐sensitivity test, and one part was used for directly DNA extraction. The information of donors and samples is further shown at Table 1.

**Table 1:** Information of donor and milk sample

Sample donor	Age	Milk type	Delivery	Children	Antibiotic
M1	26	T	CS	3	Cefazolin
M2	25	T	CS	1	Cefazolin
M3	30	T	NSD	1	Cephalexin
M4	31	T	NSD	1	Amoxicillin
M5	22	T	NSD	2	
M6	43	T	CS	2	Cefazolin
M7	28	T	CS	1	Cefazolin
M8	28	T	CS	1	Cefazolin
M9	33	T	CS	2	Cefazolin
M10	28	T	NSD	1	Cefazolin, amoxicillin
M11	26	T	CS	5	Cefazolin
M12	34	C	NSD	4	
M13	35	T	NSD	1	
M14	34	T	CS	2	Cefazolin
M15	31	M	CS	2	Cefazolin
M16	33	T	NSD	2	
M17	24	T	CS	1	Cefazolin
M18	23	T	CS	2	Cefazolin
M19	33	T	CS	3	Cefazolin
M20	19	T	CS	1	Cefazolin
M21	35	T	CS	2	Cefazolin
M22	17	C	NSD	1	
M23	32	T	CS	3	Cefazolin
M24	32	T	NSD	2	
M25	26	T	NSD	1	
M26	33	M	CS	1	Cefazolin
M27	32	T	NSD	2	
M28	32	T	CS	1	Cefazolin
M29	22	T	CS	1	Cefazolin
M30	22	T	CS	2	Cefazolin

C: colostrum, milk samples were harvested less than 3 days after delivery; T: transitional milk, milk samples were harvested between 3 and 10 days; M: mature milk, milk samples were obtained more than 10 days after delivery; NSD: normal spontaneously delivery; CS: Caesarean section.

Childern: how many kids the milk donors have. Antibiotic: milk donors were prescribed antibiotic prophylaxis.

### 2.3 Total bacterial counts, bacterial isolation, and identification

The aerobic TBCs were determined for all milk samples by following a standard plate count approach defining the bacterial counts in milk samples. Briefly, milk samples were immediately transferred to the laboratory. Each milk sample was mixed well thoroughly and a 1 ml whole milk samples was subjected to bacteria count approach. After a serial 10‐fold dilution using sodium chloride solution (0.85%), transfer 1 ml of each dilution into duplicate petri dishes. Then, add 12–15 ml plate count agar (cooled to 45 ± 1°C; tryptone 5 g, yeast extract 2.5 g, dextrose 1 g, agar 15 g, distilled water 1,000 ml, pH, 7.0 ± 0.2; prepared by autoclaving for 15 min at 121°C) on the dilution and mixing gently within 15 min of original dilution. Solidified petri dishes were then incubated under aerobic condition at 37°C for 48 hr, and we counted the number of bacterial colonies that appear on each of the plates that has between 30 and 300 colonies. TBCs (colony‐forming units/ml, CFU/ml) were determined by average of duplicated experiments.

The bacterial isolates were harvested and identified by following standard laboratory procedures, including the standard protocol for isolation of bacteria from body fluid cultures or anaerobic cultures (Chen et al., 2016). Briefly, aerobic bacterial isolates were obtained by plating samples on blood agar plates (BAPs)/eosin‐methylene blue (EMB) biplates and Chocolate agar plates for aerobic bacterial cultures. Plates were then incubated under aerobic conditions at 37°C for 24–72 hr. Anaerobic bacterial isolates were obtained by plating the samples on *Lactobacillus* de Man Rogosa and Sharpe (MRS), *Bacteroides* Bile Esculin (BBE), and kanamycin‐vancomycin laked blood (KVLB) agar plates. Plates were then incubated under anaerobic conditions at 37°C for 72 hr in a Bugbox anaerobic work station (Ruskinn Technology, Ltd., Pencoed, UK; atmospheric composition: 80% N_2_, 10% CO_2_, and 10% H_2_). For genotypic identification, chromosomal DNA of the isolates was extracted and used as a template in polymerase chain reaction (PCR), as previously described (Wang, Shyu, Ho, & Chiou, 2008), using primer sequences for the 16S rRNA gene (Wang, Shyu, Ho, & Chiou, 2007; Temmerman, Huys, & Swings, 2004). Moreover, PCR was carried out as previously described (Chen et al., 2016). Briefly, PCR was conducted in a final volume of 50 μl containing 10 mM Tris‐HCl (pH 9.0), 50 mM KCl, 1.5 mM MgCl_2_, 0.2 mM dNTP, 100 ng chromosomal DNA template, 20 pM primers, and 2 U Taq DNA polymerase (Promega, Madison, WI, USA). Thermal cycling was carried out as follows: initial denaturation at 94°C for 30 s, followed by 30 cycles at 50°C for 1 min, 72°C for 1.5 min, and 94°C for 1.5 min, and a final extension at 72°C for 5 min in a PerkinElmer GeneAmp 9600 PCR system (Applied Biosystems, Foster City, CA, USA). After checking the quality of PCR products on agarose, the PCR products are prepared for DNA Sequencing using BigDye Terminators and dGTP BigDye Terminators (Thermo Fisher, Waltham, MA, USA) and analyzed by Applied Biosystems 3730xl DNA Analyzer (Applied Biosystems, Foster City, CA, USA) according to Capillary Electrophoresis Chemistry Guide. Finally, the sequences are confirmed by BLAST searches against the GenBank database at the National Center for Biotechnology Information (http://blast.ncbi.nlm.nih.gov/Blast.cgi). As for bacterial classification, when the comparisons show more than 97% homology (concordance rate over 97%) will be identified as the same bacterial species; when the comparisons show more than 95% similarity or the concordance rate is over 97% but an overlapping to other species has been recognized, this will be only classified as genus‐level.

### 2.4 Antimicrobial susceptibility test

The antibiotic susceptibility of bacterial isolates was determined using Kirby–Bauer's disk diffusion method (Hudzicki, 2009). The results were analyzed and interpreted according to CLSI guidelines using the disk‐diffusion technique (Wikler, 2007). All antibiotic disks were purchased from Oxoid Ltd. (Oxoid, Basingstoke, UK). Oxacillin (1 μg), ampicillin (10 μg), cephalothin (30 μg), amoxicillin (25 μg), ciprofloxacin (5 μg), erythromycin (15 μg), clindamycin (2 μg), gentamicin (10 μg), and oxytetracycline (30 μg) were selected to test the antibiotic resistance of bacterial isolates. Moreover, antimicrobial susceptibility testing was routinely performed using the quality control organisms recommended by NCCLS (Wikler, 2007), including *Escherichia coli* ATCC 25922, *Staphylococcus aureus* ATCC 25923, *Pseudomonas aeruginosa* ATCC 27853, *Haemophilus influenzae* ATCC 49766, and *Streptococcus pneumoniae* ATCC49619 to validate the disk‐diffusion assay and the effectiveness of antibiotic disks. Moreover, the susceptible, intermediate, and resistant zone diameter interpretive criteria for selected antibiotics on different bacterial genera or specie could be different (Wikler, 2007), and the susceptible, intermediate, and resistant zone diameter interpretive criteria for Oxacillin (1 μg) on *S. aureu*s and *S. lugduensis* are ≥13 mm, 11–12 mm, and ≤10 mm, respectively. The susceptible, intermediate, and resistant zone diameter criteria for ampicillin (10 μg) on Enterobacteriaceae are ≥17 mm, 14–16 mm, and ≤16 mm, respectively; the susceptible and resistant zone diameter for ampicillin (10 μg) on *Staphylococcus* spp. are ≥29 mm and ≤28 mm, respectively; the susceptible and resistant zone diameter for ampicillin (10 μg) on *Enterococcus* spp. are ≥17 mm and ≤16 mm, respectively; the susceptible zone diameter for ampicillin (10 μg) on *Streptococcus* spp.β‐Hemolytic spp. are ≥17 mm and ≤16 mm, respectively. The susceptible, intermediate, and resistant zone diameter criteria for cephalothin (30 μg) on Enterobacteriaceae and *Staphylococcus* spp. are ≥18 mm, 15–17 mm, and ≤14 mm, respectively. The susceptible, intermediate, and resistant zone diameter criteria for ciprofloxacin (5 μg) on Enterobacteriaceae, *Acinetobacter*,* Staphylococcus* spp., and *Enterococcus* spp. are ≥21 mm, 16–20 mm, and ≤15 mm, respectively. The susceptible, intermediate, and resistant zone diameter for erythromycin (15 μg) on *Staphylococcus* spp. and *Enterococcus* spp. are ≥23 mm, 14–22 mm, and ≤13 mm, respectively; the susceptible, intermediate, and resistant zone diameter for erythromycin (15 μg) on *streptococcus* spp. are ≥21 mm, 16–20 mm, and ≤15 mm, respectively. The susceptible, intermediate, and resistant zone diameter for clindamycin (2 μg) on *Staphyloccoccus* spp. are ≥21 mm, 15–20 mm, and ≤14 mm, respectively; the susceptible, intermediate, and resistant zone diameter for clindamycin (2 μg) on *Streptococcus* spp. Viridans group are ≥19 mm, 16–18 mm, and ≤15 mm, respectively. The susceptible, intermediate, and resistant zone diameter criteria for gentamicin (10 μg) on Enterobacteriaceae, *Acinetobacter* spp. and *Staphyloccoccus* spp. are ≥15 mm, 13–14 mm, and ≤12 mm, respectively. The susceptible, intermediate, and resistant zone diameter criteria for tetracycline (30 μg) on Enterobacteriaceae and *Acinetobacter* spp. are ≥15 mm, 12–14 mm, and ≤11 mm, respectively. The inhibition zones had been measured twice with a ruler, and only the smallest zone had been recognized. If we cannot determine the range of inhibition zone, we had retested that disks 1–2 times. The multidrug‐resistant bacterial strains have been defined accordingly to a previous report. Briefly, when the clinical isolates were resistant to at least one antimicrobial agent in three or more antimicrobial categories, these isolates were defined as multidrug‐resistant bacteria (Magiorakos et al., 2012).

## 3 RESULTS AND DISCUSSIONS

### 3.1 Sample information and bacterial isolates in human milk

Table 2 shows information regarding the isolated bacterial strains in milk samples. All mothers recruited in this study did not have clinical mastitis, fever, discomfort, or other clinical symptoms. Moreover, no redness, pain, or lumps were observed in their breasts. As shown in Table 2 bacterial isolates were identified among all milk samples, and most bacterial isolates in milk are well‐known members of the human microbiota and some of them are opportunistic pathogens (Fernandez et al., 2013). Moreover, the most prevalent or common bacterial isolate in milk samples was *S. epidermidis*, which was isolated in 26 samples. Notably, a *S. aureus* strain was isolated in five milk samples (M8, M9, M12, M17, and M22). The *S. aureus* has long been recognized as an important etiological pathogen in human disease but carriers of *S. aureus* in their microbiota are almost 20% of the healthy population. Accordingly, these isolates are not obligatory pathogenic (Kluytmans, van Belkum, & Verbrugh, 1997). It should be indicated that the bacterial counts within samples should play much more important roles in pathogenicity. For instance, the total bacterial counts in milk samples have been reported to serve as indicators of herd udder health (Jayarao, Pillai, Sawant, Wolfgang, & Hegde, 2004; Hayes et al., 2001). Despite the wide distributions of the above two bacterial strains in many milk samples in this study, these bacteria did not appear to cause harm to their hosts or the breast‐fed babies because no milk donors suffered from clinical mastitis, as described above. However, as much higher bacterial counts were found in several milk samples (described below), the roles of these *S. aureus* in milk should be further investigated.

**Table 2:** Bacterial isolates from human milk

Samples	Milk bacterial isolates
M1	*Streptococcus parasanguinis, Staphylococcus epidermidis, Propionibacterium acnes*
M2	*Enterococcus faecalis, Staphylococcus epidermidis*
M3	*Staphylococcus epidermidis, Enterococcus faecaliss*
M4	*Staphylococcus epidermidis, Streptococcus salivarius*
M5	*Staphylococcus epidermidis, Acinetobacter ursingii septica*
M6	*Staphylococcus epidermidis, Streptococcus lactarius, Staphylococcus hominis*
M7	*Enterobacter aerogenes, Enterococcus faecalis*
M8	*Staphylococcus epidermidis, Staphylococcus aureus*
M9	*Staphylococcus aureus, Staphylococcus epidermidis, Rothia mucilaginosa*
M10	*Micrococcus luteus, Staphylococcus lugdunensis, Staphylococcus epidermidis, Pseudomonas oryzihabitans*
M11	*Staphylococcus lugdunensis, Staphylococcus epidermidis*
M12	*Staphylococcus epidermidis, Staphylococcus aureus, Moraxella osloensis, Enhydrobacter aerosaccus*
M13	*Staphylococcus epidermidis, Pseudomonas monteilii*
M14	*Rothia dentocariosa, Streptococcus parasanguinis, Streptococcus mitis, Streptococcus oralis*
M15	*Staphylococcus epidermidis, Corynebacterium kroppenstedtii, Staphylococcus lugdunensis, Corynebacterium kroppenstedtii*
M16	*Staphylococcus epidermidis, Staphylococcus lugdunensis*
M17	*Staphylococcus lugdunensis, Staphylococcus aureus, Staphylococcus epidermidis, Enterococcus faecalis, Corynebacterium kroppenstedtii*
M18	*Stayphylococcus hominis, Acinetobacter calcoaceticus, Staphylococcus epidermidis, Corynebacterium kroppenstedtii*
M19	*Staphylococcus epidermidis, Rothia mucilaginosa, Streptococcus* sp.
M20	*Acinetobacter* sp.*, Staphylococcus hominis, Acinetobacter calcoaceticus*
M21	*Acinetobacter* sp.*, Staphylococcus epidermidis, Staphylococcus lugdunensis, Streptococcus lactarius, Streptococcus* sp.*, Corynebacterium kroppenstedtii*
M22	*Staphylococcus epidermidis, Corynebacterium striatum, Corynebacterium jeikeium, Enterococcus faecalis, Staphylococcus aureus*
M23	*Staphylococcus epidermidis, Corynebacterium simulans*
M24	*Staphylococcus hominis, Staphylococcus lugdunensis, Streptomyces* sp.*, Rothia dentocariosa, Streptococcus parasanguinis*
M25	*Staphylococcus epidermidis, Staphylococcus hominis*
M26	*Staphylococcus epidermidis*
M27	*Staphylococcus haemolyticus, Staphylococcus epidermidis, Staphylococcus lugdunensis*
M28	*Rothia mucilaginosa, Staphylococcus epidermidis*
M29	*Staphylococcus* sp.*, Staphylococcus epidermidis*
M30	*Staphylococcus epidermidis*

### 3.2 Total bacterial counts in milk samples

**Figure 1:**
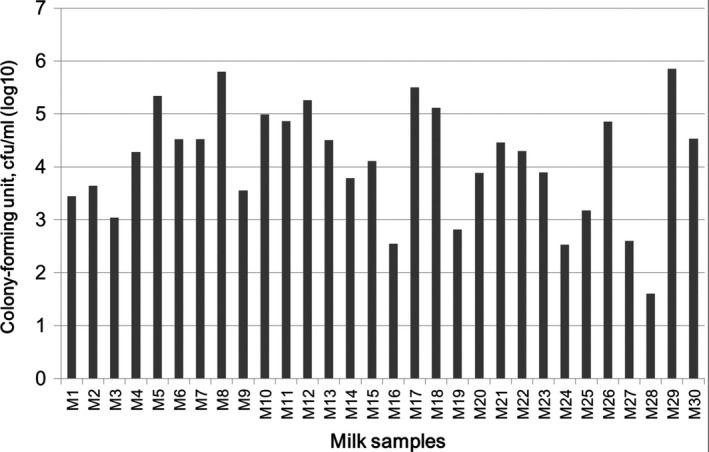
Mean aerobic total bacterial count in milk samples. Each sample was assayed by standard plate count method, and the solid bars represent the mean value from duplicated experiments

In this study, we also evaluated the characteristics of milk samples by determining the total bacterial counts (TBCs) in milk samples from healthy donors. To date, there are no regulations regarding the TBC criteria in human milk to restrict mothers from breastfeeding their babies. However, the bacterial counts have been evaluated in bulk tank milk or in raw milk from dairy farms, and the bacterial counts have been shown to serve as indicators and facilitate monitoring of herd udder health and milk quality (Jayarao et al., 2004; Hayes et al., 2001). In contrast, with regarding to human milk sharing or milk samples collected by milk banks, several regulations have been employed to evaluate the milk safety (Balmer & Wharton, 1992). For reference, milk samples with counts less than 10^3^ CFU/ml are considered acceptable, regardless of the organisms present, and milk with counts more than 10^5^ CFU/ml cannot be used (Balmer & Wharton, 1992). Moreover, when donor milk has bacterial counts between 10^3^ and 10^5^ CFU/ml, it is only accepted if the organisms are skin commensals, such as *Staphylococcus epidermidis*, viridans streptococci, and diphtheroids (Balmer & Wharton, 1992). Notably, donor milk is not accepted if TBCs are more than 10^3^ CFU/ml with *S. aureus*, any gram‐negative rod, β hemolytic *Streptococci*, or *E. faecalis* (Balmer & Wharton, 1992; Ng, Lee, Leung, Wong, & Ho, 2004). In this study, the TBCs in milk samples ranged from 10^1^ to 10^6^ CFU/ml (Figure 1), indicating a wide distribution of TBCs within milk samples. Furthermore, according to the TBC criteria for milk sharing above, about 22 samples (73% acceptance) could be considered suitable for further milk sharing or for collection by milk banks. As indicated above, *S. aureus* was isolated in M8, M9, M12, M17, and M22 samples. Thus, according to the TBC criteria above, these five milk samples were not suitable for milk sharing. Despite this, all milk donors were healthy during sampling. They did not show any symptoms related to clinical mastitis. Thus, the *S. aureus* in these five non‐accepted milk samples did not appear to lead to infection regardless of their TBCs. Furthermore, although totally eight milk samples were not considered suitable for milk sharing due to the TBC criteria, all milk donors in the current study could still breastfeed their own infants for several reasons. First, there are no TBC criteria limiting mothers from breastfeeding their own infants in Taiwan or in other countries. Second, it is well documented that even mothers suffering from mastitis can still breastfed their own babies, and only mothers infected with human immunodeficiency virus (Xia et al., 2014; Wright et al., 2014), cytomegalovirus (Wiemken et al., 2014; Wang et al., 2014), or human T‐cell leukemia virus type I (Li et al., 2004) should not breastfeed their own babies (Lawrence & Lawrence, 2001). Collectively, although a wide spectrum of TBCs (Figure 1) and various bacterial isolates (Table 2) were observed in milk samples from healthy mothers, these milk samples were still considered safe, and all milk donors could still breastfeed their own infants regardless of TBC levels in milk as described above. In contrast, when milk samples are donated for preterm infants or milk banks, only 73% milk samples could be accepted by the milk banks, and the high values of TBC and potential pathogens, such as *S. aureus*, should be considered. We speculated that the high levels of TBCs in several milk samples may play a role in supplying more bacterial strains to the infant gut of healthy babies, as suggested previously (Fernandez et al., 2013; Solis et al., 2010).

### 3.3 Antibiotic susceptibility of bacterial isolates

**Figure 2:**
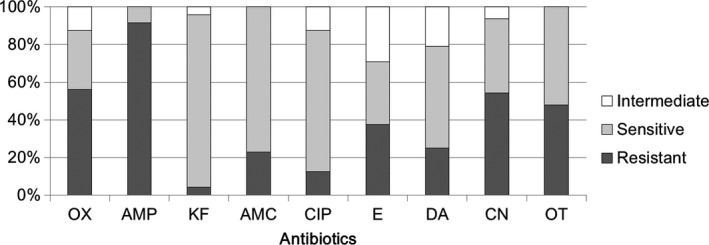
Comparative efficacies of antibiotics for the treatment of *Staphylococcus* spp. of human milk. Antibiotic susceptibility analysis was performed on milk‐isolated *Staphylococcus* spp. (totally 48 strains) toward different antibiotics. The ratio for intermediate resistant (intermediate), sensitive, and resistant efficacies were shown. OX, Oxacillin, AMP, Ampicillin, KF, Cephalothin, AMC, Amoxicillin, CIP, Ciprofloxacin, E, Erythromycin, DA, Clindamycin, CN, Gentamicin, OT, Oxytetracycline

To monitor antibiotic susceptibility of bacterial isolates from human milk samples of healthy donors, five milk samples were collected each month, and a total of 30 milk samples were harvested during a 6 months period. The antibiotic sensitivities of bacterial strains isolated from milk samples are indicated in Supplementary Tables S1–S4. Supplementary Table S1 shows the antibiotic susceptibilities of *Staphylococcus*‐related isolates. Nine antibiotics were selected as test drugs because these antibiotics are able to inhibit the growth of susceptible *Staphylococcus* spp. in the clinical setting. Our data showed that *Staphylococcus* strains found in milk resisted to 1–8 of the selected antibiotics. Totally, these *Staphylococcus* strains resisted to 48.6 ± 20.1% selected antibiotics. The efficacies of each selected antibiotic against the growth of all *Staphylococcus* isolates were also calculated (Figure 2). The results showed a high rate of antibiotic resistance of *Staphylococcus* to ampicillin (91%), oxacillin (56%), gentamicin (54%), and oxytetracycline (48%). Moreover, higher rates of antibiotic sensitivity of *Staphylococcus* to cephalothin (92%), amoxicillin (77%), and ciprofloxacin (75%) were observed. Notably, as just mentioned, *Staphylococcus* were exhibited 56% resistant to oxacillin (equal to resistant to methicillin), implying that this *S. aureus* in M12 samples should be Methicillin‐resistant *Staphylococcus aureus* (MRSA) strain. On the other hand, several different definitions for multidrug‐resistant bacteria have been used to characterize the patterns of resistance of bacterial isolates. For instance, a recent study indicates that when the clinical isolates were resistant to at least one antimicrobial agent in three or more antimicrobial categories, and these isolates could be defined as multidrug‐resistant bacteria (Magiorakos et al., 2012). Thus, the majority of the isolated strains (25/48) in Supplementary Table S1 are multiresistant strains according to the definition above.

Previous studies have reported strongly antibiotic‐resistant *Staphylococcus* strains from human milk of healthy donors (Begovic et al., 2013; Carneiro, Queiroz, & Merquior, 2004). For example, high antibiotic‐resistance rates of *S. epidermidis*,* S. warneri*,* S. haemolyticus*, and *S. aureus* isolated from milk to penicillin (87%) and erythromycin (59.3%) have been observed. Moreover, several *S. epidermidis* isolates harvested from human milk have been shown to be resistant to tetracycline, erythromycin, clindamycin, and vancomycin (Begovic et al., 2013). In our previous study, most *Staphylococcus* spp. isolates in milk were also shown to display resistance to multiple antibiotics (Chen et al., 2016). In this study, the majority of the isolated strains are still multiresistant strains. These bacteria could play a role in mediating the risk of antibiotic resistance in milk samples.

**Figure 3:**
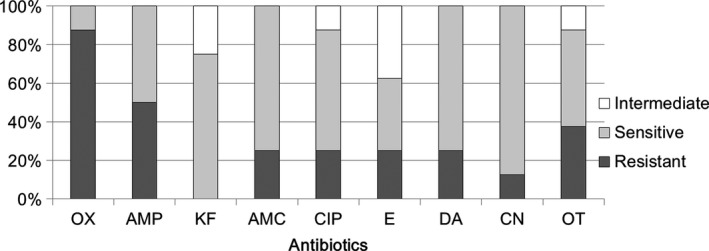
Comparative efficacy of antibiotics for the treatment of *Streptococcus* spp. of human milk. Antibiotic susceptibility for *Streptococcus* spp. (totally eight strains) toward different antibiotics. OX, Oxacillin, AMP, Ampicillin, KF, Cephalothin, AMC, Amoxicillin, CIP, Ciprofloxacin, E, Erythromycin, DA, Clindamycin, CN, Gentamicin, OT, Oxytetracycline

In our previous report, *Streptococcus* spp. was isolated only in three milk samples, and these isolates displayed strong antibiotic resistance to selected antibiotics (Chen et al., 2016). In this study, a total of seven milk samples harbored *Streptococcus*‐related isolates (Supplementary Table S2), and two strains out of eight exhibited resistance to one antimicrobial agent in three or more antimicrobial classes as defined previously (multiresistant) (Magiorakos et al., 2012). Additionally, high antibiotic‐sensitivity rates of *Streptococcus* to cephalothin (75%), amoxicillin (75%), ciprofloxacin (63%), clindamycin (75%), and gentamicin (88%) were observed. However, our data also showed high rates of antibiotic resistance to oxacillin (88%) and ampicillin (50%) in these isolates (Figure 3). Totally, they resisted to 41.7 ± 26.4% selected antibiotics.

**Figure 4:**
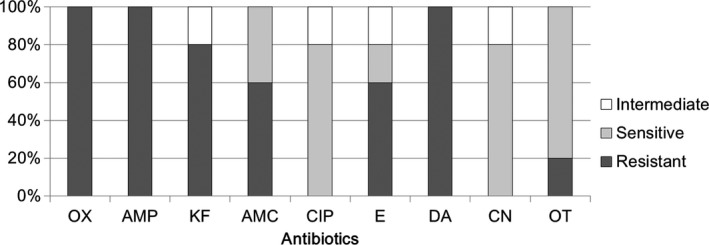
Comparative efficacy of antibiotics for the treatment of *Acinetobacter* spp. of human milk. Antibiotic susceptibility analysis was performed on milk‐isolated *Acinetobacter* spp. (totally five strains) toward different antibiotics. The ratio for intermediate resistant (intermediate), sensitive, and resistant efficacies were shown. OX, Oxacillin, AMP, Ampicillin, KF, Cephalothin, AMC, Amoxicillin, CIP, Ciprofloxacin, E, Erythromycin, DA, Clindamycin, CN, Gentamicin, OT, Oxytetracycline

Supplementary Table S3 shows the antibiotic sensitivities of *Acinetobacter* spp. and *Enterococcus* spp. from human milk to all tested antibiotics. *Acinetobacter* isolates were resistant to 66.7 ± 13.6% tested antibiotics, and *Enterococcus* isolates were resistant to 73.3 ± 6.1% tested antibiotics. Figure 4 shows the rates of antibiotic sensitivity of *Acinetobacter* to three antibiotics, that is, ciprofloxacin (80%), gentamicin (80%), and oxytetracycline (80%). Notably, high rates of antibiotic resistance of *Acinetobacter* to six antibiotics, that is, oxacillin (100%), ampicillin (100%), clindamycin (100%), cephalothin (80%), amoxicillin (60%), and erythromycin (60%), were observed. *Acinetobacter* is a gram‐negative coccobacillus that has been recently recognized as an infectious agent of importance to hospitals worldwide (Fournier & Richet, 2006). Among *Acinetobacter* spp., *A. baumannii* has been shown to be an important pathogen in healthcare‐associated infections. This species commonly shows resistance to multiple antibiotics and is difficult to treat (Fishbain & Peleg, 2010; Michalopoulos & Falagas, 2010). In this study, no *A. baumannii* was isolated from milk samples. However, as indicated above, the other *Acinetobacter*‐related isolates were resistant to six antibiotics, indicating that these *Acinetobacter* species may play a role in maintaining severe antibiotic resistance in human milk.

**Figure 5:**
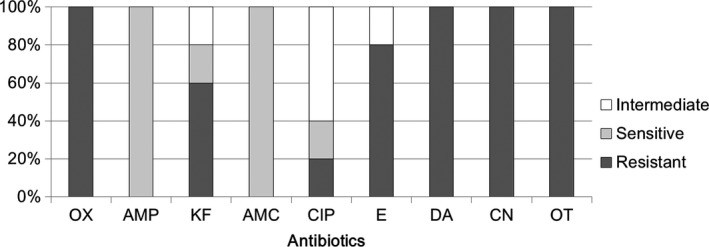
Comparative efficacy of antibiotics for the treatment of *Enterococcus* spp. of human milk. Antibiotic susceptibility analysis was performed on milk‐isolated *Enterococcus* spp. (totally six strains) toward different antibiotics. The ratio for intermediate resistant (intermediate), sensitive, and resistant efficacies were shown. OX, Oxacillin, AMP, Ampicillin, KF, Cephalothin, AMC, Amoxicillin, CIP, Ciprofloxacin, E, Erythromycin, DA, Clindamycin, CN, Gentamicin, OT, Oxytetracycline

The presence of *E. faecium* and *E. faecalis* in milk of healthy women has been reported previously (Hunt et al., 2011; Reviriego et al., 2005; Jimenez et al., 2008), and a recent report also found that *E. faecalis*,* E. faecium*,* E. hirae*,* E. casseliflavus*, or *E. durans* were distributed in milk samples from healthy pigs, dogs, sheep, cats, and humans. Moreover, most *Enterococcus* spp. cultured from milk of healthy women showed resistance to various clinically relevant antibiotics (Jimenez et al., 2013). In Figure 5, *Enterococcus* spp. were sensitive to ampicillin (100%) and amoxicillin (100%), but exhibited 100% resistance to oxacillin, clindamycin, gentamicin, and Oxytetracycline, and partial resistance to cephalothin (60%) and erythromycin (80%). These data suggested that *Enterococcus* spp. and *Acinetobacter* could more likely to confer a strong risk of antibiotic resistance in human milk samples worldwide. Another study also found a wide distribution of virulence genes and/or antibiotic resistance among the *E. faecalis* and *E. faecium* isolated from human milk (Jimenez et al., 2013). This previous study suggests that above bacterial strains could play roles in maintaining an antibiotic‐resistant reservoir in animals and humans (Jimenez et al., 2013). Thus, further studies are needed to determine whether all *Enterococcus* spp. isolated from milk may contain virulence genes.

**Figure 6:**
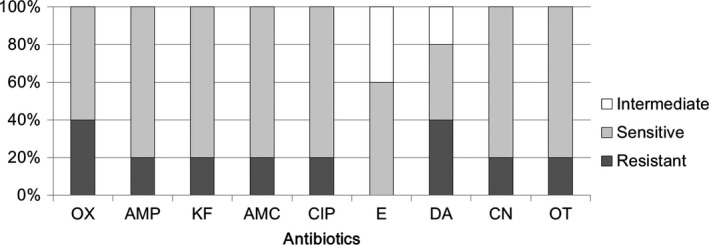
Comparative efficacy of antibiotics for the treatment of *Corynebacterium* spp. of human milk. Antibiotic susceptibility analysis was performed on milk‐isolated *Corynebacterium* spp. (totally five strains) toward different antibiotics. The ratio for intermediate resistant (intermediate), sensitive, and resistant efficacies were shown. OX, Oxacillin, AMP, Ampicillin, KF, Cephalothin, AMC, Amoxicillin, CIP, Ciprofloxacin, E, Erythromycin, DA, Clindamycin, CN, Gentamicin, OT, Oxytetracycline

**Figure 7:**
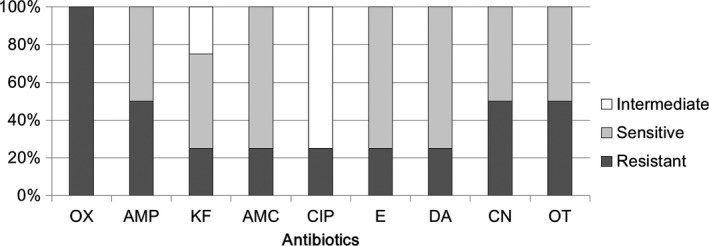
Comparative efficacy of antibiotics for the treatment of *Rothia* spp. of human milk. Antibiotic susceptibility analysis was performed on milk‐isolated *Rothia* spp. (totally four strains) toward different antibiotics. The ratio for intermediate resistant (intermediate), sensitive, and resistant efficacies were shown. OX, Oxacillin, AMP, Ampicillin, KF, Cephalothin, AMC, Amoxicillin, CIP, Ciprofloxacin, E, Erythromycin, DA, Clindamycin, CN, Gentamicin, OT, Oxytetracycline

Supplementary Table S4 shows the antibiotic sensitivity testing for *Corynebacterium* spp. of human milk. Nine antibiotics were selected because these antibiotics are able to inhibit the growth of susceptible *Corynebacterium* spp. and *Rothia* spp. strains in the clinical setting. Among five *Corynebacterium* isolates, one isolate from M22 was resistant to almost all selected antibiotics, whereas the other *Corynebacterium* isolates were sensitive to 66%–100% of selected antibiotics. Furthermore, we observed high rates of antibiotic sensitivity (80%) of *Corynebacterium* to ampicillin, cephalothin, amoxicillin, ciprofloxacin, gentamicin, and oxytetracycline and moderate rates (60%) of sensitivity to oxacillin and erythromycin (Figure 6). Thus, these data demonstrated that most *Corynebacterium* isolates from milk samples were sensitive to clinically relevant antibiotics. Moreover, *Rothia*‐related isolates were resistant to 58.2 ± 31.9% of tested antibiotics. Additionally, these bacterial isolates were resistant to oxacillin (100%), ampicillin (50%), gentamicin (50%), and oxytetracycline (50%), but sensitive to amoxicillin (75%), erythromycin (75%), and clindamycin (75%), as shown in Figure 7. These isolates resisted to at least one agent in three or more than three antimicrobial categories. As the result, they were multiresistant strains (4 multiresistant strains/4) according to a recent definition (Magiorakos et al., 2012).

In this study, 22 milk donors were treated with cephalexin, amoxicillin or cefazolin for antibiotic prophylaxis (Table 1). Both cephalexin and cefazolin are first generation cephalosporins but cephalexin is available orally where cefazolin is available as injection. According to the results from Supplementary Table S1 to S4, antibiotic prophylaxis received by milk donors M1, M2, M3, M4, M6, M8, M9, M14, M15, M19, M23, M26, M29 and M30 could be active against the milk‐isolated bacterial strains. But cephalothin is not active against the Enterococcus‐related strains from M7, and amoxicillin is not active against the *S. lugdunensis* from M10; moreover, cephalothin is not active against *S. epidermidis* and *E. faecalis* which were isolated from M17, and it has no activity against *Acinetobacter*‐related bacteria isolated from M18, M20, and M21 (Supplementary Table S3), and this antibiotic is not active against *R. mucilaginosa* from M28 (Supplementary Table S4). These findings indicate that cephalothin or cefazolin prophylaxis may not be active enough against all milk‐isolated bacterial strains from several milk donors. Doctors often prescribe cephalexin or cefazolin prophylaxis in this hospital, and as a result, these antibiotic prophylaxes may play some roles in elevating the antibiotic *Acinetobacter* spp. but this should be further investigated. In our previous pilot study, only 19 human donors had been recruited but we have observed some mild to strong antibiotic resistance among bacterial strains in human milk from healthy donors. Our current observation had confirmed that most bacterial strains in human milk could be multiresistant strains. Whether this is a prevalent situation in the other milk samples from the other areas or hospitals in Taiwan should be further investigated.

In conclusion, this study reported the bacterial counts and the antibiotic susceptibility pattern of commensally bacteria in human milk from healthy mothers in Taiwan. The majority of the isolated bacterial strains from human milk samples are multiresistant strains. Otherwise, the bacterial counts ranged from 4.0 × 10^1^ to 7.1 × 10^5^ CFU/ml, implying a wide spectrum of bacteria in milk samples from healthy mothers. Refer to the previously described total bacterial counts criteria for the use of donated human milk, only 73% of the current milk samples could be accepted for the milk bank. Although a wide spectrum of TBCs and the various bacterial patterns had been observed in milk samples from healthy milk donors but no milk donors suffered from any infections or symptoms of mastitis. These indicated that these diverse commensal bacteria in milk did not harm their hosts. In milk samples for preterm infants, higher TBCs levels in some milk samples should be kept in mind, and the roles of these antibiotic‐resistant bacterial isolates in milk from healthy donors should be further investigated.

## CONFLICT OF INTEREST

No conflict of interest exists.
