# Supplementary material for: Most commensally bacterial strains in human milk of healthy mothers display multiple antibiotic resistance
Source: Microbiologyopen. 2018 Mar 25;8(1):e00618. doi: 10.1002/mbo3.618 (PMC6341030; doi:10.1002/mbo3.618)
Supplement: Supplementary file 1 [file MBO3-8-e00618-s001.docx]

**Supplementary** **Table S3.** Antibiotic sensitivity testing for *Staphylococcus* spp. of human milk.

|  |  | Antibiotics | | | | | | | | |
| --- | --- | --- | --- | --- | --- | --- | --- | --- | --- | --- |
| Sample |  | OX^a^ | AMP | KF | AMC | CIP | E | DA | CN | OT |
| M1 | *Staphylococcus epidermidis* | S^b^ | R^b^ | S | S | S | R | S | S | S |
| M2 | *Staphylococcus epidermidis* | I^b^ | R | S | S | S | R | S | S | S |
| M3 | *Staphylococcus epidermidis* | I | R | S | S | S | S | S | R | R |
| M4 | *Staphylococcus epidermidis* | R | R | S | S | S | R | I | R | R |
| M5 | *Staphylococcus epidermidis* | I | R | S | S | S | R | R | R | S |
| M6 | *Staphylococcus epidermidis* | R | R | S | S | R | R | R | I | S |
| M6 | *Staphylococcus hominis* | R | R | S | S | R | R | S | R | S |
| M8 | *Staphylococcus aureus* | I | R | S | S | S | I | S | S | R |
| M8 | *Staphylococcus epidermidis* | R | R | S | S | S | S | I | R | S |
| M9 | *Staphylococcus aureus* | S | R | S | S | S | I | S | S | R |
| M9 | *Staphylococcus epidermidis* | I | R | S | S | S | S | S | S | S |
| M10 | *Staphylococcus lugdunensis* | R | R | I | R | S | S | S | R | R |
| M10 | *Staphylococcus epidermidis* | R | R | S | S | S | I | R | S | R |
| M11 | *Staphylococcus lugdunensis* | S | S | S | S | I | I | I | S | S |
| M11 | *Staphylococcus epidermidis* | R | R | S | S | S | I | R | R | R |
| M12 | *Staphylococcus epidermidis* | R | R | S | S | S | S | I | R | R |
| M12 | *Staphylococcus aureus* | R | R | S | R | I | I | S | R | R |
| M13 | *Staphylococcus epidermidis* | S | R | S | S | I | S | S | R | S |
| M15 | *Staphylococcus epidermidis* | S | R | S | S | S | S | S | R | S |
| M15 | *Staphylococcus lugdunensis* | S | R | S | S | S | S | S | R | R |
| M16 | *Staphylococcus epidermidis* | R | R | S | R | S | I | S | S | R |
| M16 | *Staphylococcus lugdunensis* | R | R | S | R | S | S | S | R | R |
| M17 | *Staphylococcus lugdunensis* | I | R | S | S | S | S | S | S | S |
| M17 | *Staphylococcus aureus* | S | R | S | S | I | I | I | S | S |
| M17 | *Staphylococcus epidermidis* | R | R | R | S | I | R | R | I | R |
| M18 | *Staphylococcus epidermidis* | S | R | S | S | S | R | R | R | S |
| M19 | *Staphylococcus epidermidis* | R | R | S | R | S | R | I | R | R |
| M20 | *Staphylococcus hominis* | R | R | S | R | S | I | S | S | S |
| M20 | *Staphylococcus hominis* | S | R | S | S | S | R | S | I | R |
| M21 | *Staphylococcus epidermidis* | S | R | S | S | S | R | S | R | R |
| M21 | *Staphylococcus lugdunensis* | R | R | S | R | S | S | S | R | R |
| M22 | *Staphylococcus epidermidis* | R | R | S | R | S | I | S | S | R |
| M22 | *Staphylococcus epidermidis* | R | R | S | S | S | I | S | S | R |
| M22 | *Staphylococcus aureus* | S | R | S | S | S | I | S | S | S |
| M23 | *Staphylococcus epidermidis* | R | R | S | S | S | S | R | R | S |
| M23 | *Staphylococcus epidermidis* | R | R | S | S | S | I | I | R | R |
| M24 | *Staphylococcus hominis* | S | S | S | S | S | R | S | S | S |
| M24 | *Staphylococcus lugdunensis* | R | R | S | S | S | S | S | R | S |
| M25 | *Staphylococcus epidermidis* | S | S | S | S | R | R | S | R | S |
| M25 | *Staphylococcus hominis* | R | R | S | S | R | R | R | R | R |
| M26 | *Staphylococcus epidermidis* | S | R | S | S | S | S | S | S | S |
| M26 | *Staphylococcus epidermidis* | S | S | S | S | S | S | R | S | S |
| M27 | *Staphylococcus haemolyticus* | R | R | R | R | R | R | R | R | S |
| M27 | *Staphylococcus epidermidis* | R | R | I | R | S | S | I | S | S |
| M28 | *Staphylococcus epidermidis* | R | R | S | S | R | R | R | R | R |
| M29 | *Staphylococcus epidermidis* | R | R | S | R | S | R | R | S | S |
| M30 | *Staphylococcus epidermidis* | R | R | S | S | S | R | I | R | R |
| M30 | *Staphylococcus epidermidis* | R | R | S | S | I | I | I | R | S |

^a^OX: Oxacillin, AMP: Ampicillin, KF: Cephalothin, AMC: Amoxicillin, CIP: Ciprofloxacin, E: Erythromycin, DA: Clindamycin, CN: Gentamicin, OT: Oxytetracycline.

^b^S: Sensitive; R: resistant; I: Intermediate, the diameter of the zone of inhibition around a particular antibiotic ranged between the criteria of “susceptible” and “resistant”. This indicates either a technical problem that should be resolved by repeat testing or a lack of clinical experience in treating organisms with these zones [[21](#_ENREF_21)].

**Supplementary** **Table S4.** Antibiotic sensitivity testing for *Streptococcus* spp. of human milk.

|  |  | Antibiotics | | | | | | | | |
| --- | --- | --- | --- | --- | --- | --- | --- | --- | --- | --- |
| Sample |  | OX^a^ | AMP | KF | AMC | CIP | E | DA | CN | OT |
| M1 | *Streptococcus parasanguinis* | R^b^ | S^b^ | S | S | S | S | S | S | S |
| M4 | *Streptococcus salivarius* | R | R | S | S | R | R | R | R | I^b^ |
| M6 | *Streptococcus lactarius* | R | S | S | S | S | R | R | S | S |
| M14 | *Streptococcus parasanguinis* | R | S | S | S | S | S | S | S | R |
| M19 | *Streptococcus sp* | R | R | S | S | R | I | S | S | S |
| M21 | *Streptococcus lactarius* | R | R | I | R | S | I | S | S | R |
| M21 | *Streptococcus sp* | R | R | I | R | S | I | S | S | R |
| M24 | *Streptococcus sp* | S | S | S | S | I | S | S | S | S |

^a^OX: Oxacillin, AMP: Ampicillin, KF: Cephalothin, AMC: Amoxicillin, CIP: Ciprofloxacin, E: Erythromycin, DA: Clindamycin, CN: Gentamicin, OT: Oxytetracycline.

^b^S: Sensitive; R: resistant; I: Intermediate, the diameter of the zone of inhibition around a particular antibiotic ranged between the criteria of “susceptible” and “resistant”. This indicates either a technical problem that should be resolved by repeat testing or a lack of clinical experience in treating organisms with these zones [[21](#_ENREF_21)].

**Supplementary** **Table S5.** Antibiotic sensitivity testing for *Acinetobacter* spp., *Enterococcus spp.* and *Enterobacter* of human milk.

|  |  | Antibiotics | | | | | | | | |
| --- | --- | --- | --- | --- | --- | --- | --- | --- | --- | --- |
| Sample |  | OX^a^ | AMP | KF | AMC | CIP | E | DA | CN | OT |
| M5 | *Acinetobacter ursingii* | R^b^ | R | R | S^b^ | S | R | R | S | S |
| M18 | *Acinetobacter calcoaceticus* | R | R | R | R | S | I^b^ | R | S | S |
| M20 | *Acinetobacter sp* | R | R | I | S | S | S | R | I | S |
| M20 | *Acinetobacter calcoaceticus* | R | R | R | R | S | R | R | S | S |
| M21 | *Acinetobacter sp* | R | R | R | R | I | R | R | S | R |
|  |  |  |  |  |  |  |  |  |  |  |
| M2 | *Enterococcus faecalis* | R | S | I | S | S | R | R | R | R |
| M3 | *Enterococcus faecalis* | R | S | S | S | I | R | R | R | R |
| M7 | *Enterococcus faecalis* | R | S | R | S | I | R | R | R | R |
| M17 | *Enterococcus faecalis* | R | S | R | S | I | R | R | R | R |
| M22 | *Enterococcus faecalis* | R | S | R | S | R | I | R | R | R |
|  |  |  |  |  |  |  |  |  |  |  |
| M7 | *Enterobacter aerogenes* | R | I | R | R | S | R | R | S | S |

^a^OX: Oxacillin, AMP: Ampicillin, KF: Cephalothin, AMC: Amoxicillin, CIP: Ciprofloxacin, E: Erythromycin, DA: Clindamycin, CN: Gentamicin, OT: Oxytetracycline.

^b^S: Sensitive; R: resistant; I: Intermediate, the diameter of the zone of inhibition around a particular antibiotic ranged between the criteria of “susceptible” and “resistant”. This indicates either a technical problem that should be resolved by repeat testing or a lack of clinical experience in treating organisms with these zones [[21](#_ENREF_21)].

**Supplementary** **Table S6.** Antibiotic sensitivity testing for *Corynebacterium* spp. and *Rothia* spp. of human milk.

|  |  | Antibiotics | | | | | | | | |  |
| --- | --- | --- | --- | --- | --- | --- | --- | --- | --- | --- | --- |
| Sample |  | OX^a^ | AMP | KF | AMC | CIP | E | DA | CN | OT |  |
| M1 | *Corynebacterium xerosis* | S^b^ | S | S | S | S | S | I^b^ | S | S |  |
| M15 | *Corynebacterium kroppenstedtii* | S | S | S | S | S | S | S | S | S |  |
| M15 | *Corynebacterium kroppenstedtii* | S | S | S | S | S | S | S | S | S |  |
| M22 | *Corynebacterium striatum* | R^b^ | S | S | S | S | I | R | S | S |  |
| M22 | *Corynebacterium jeikeium* | R | R | R | R | R | I | R | R | R |  |
|  |  |  |  |  |  |  |  |  |  |  |  |
| M9 | *Rothia mucilaginosa* | R | S | I | S | I | S | S | S | R |  |
| M14 | *Rothia mucilaginosa* | R | R | S | S | I | S | S | S | S |  |
| M24 | *Rothia dentocariosa* | R | S | S | S | I | S | S | R | S |  |
| M28 | *Rothia mucilaginosa* | R | R | R | R | R | R | R | R | R |  |

^a^OX: Oxacillin, AMP: Ampicillin, KF: Cephalothin, AMC: Amoxicillin, CIP: Ciprofloxacin, E: Erythromycin, DA: Clindamycin, CN: Gentamicin, OT: Oxytetracycline.

^b^S: Sensitive; R: resistant; I: Intermediate, the diameter of the zone of inhibition around a particular antibiotic ranged between the criteria of “susceptible” and “resistant”. This indicates either a technical problem that should be resolved by repeat testing or a lack of clinical experience in treating organisms with these zones [[21](#_ENREF_21)].
